# Comparative effectiveness of water-based versus land-based rehabilitation in COPD: a systematic review and network meta-analysis of randomized controlled trials

**DOI:** 10.1038/s41533-026-00503-8

**Published:** 2026-04-11

**Authors:** Eleuterio A. Sánchez Romero, Ada Jacqueline Zubercová, Amélie Gran, Raphael Martins de Abreu, Francisco Xavier de Araujo, Pierluigi Sinatti, Juan Nicolás Cuenca-Zaldívar, Rob Sillevis, Camilo Corbellini

**Affiliations:** 1 Interdisciplinary Research Group on Musculoskeletal Disorders, 28014 Madrid, Spain; 2 Research Group in Nursing and Health Care, Puerta de Hierro Health Research Institute–Segovia de Arana (IDIPHISA), 28222 Majadahonda, Spain; 3 Department of Rehabilitation Sciences, Florida Gulf Coast University, Fort Myers, FL 33965 USA; 4 Physiotherapy and Orofacial Pain Working Group, Sociedad Española de Disfunción Craneomandibular y Dolor Orofacial (SEDCYDO), 28009 Madrid, Spain; 5 LUNEX University of Applied Sciences, 50 Avenue du Parc des Sports, 4671 Differdange, Luxembourg; 6 Luxembourg Health & Sport Sciences Research Institute A.s.b.l., 50 Avenue du Parc des Sports, 4671 Differdange, Luxembourg; 7 Federal University of Pelotas, R. Gomes Carneiro, 01 – Balsa, Pelotas, RS 96010-610 Brazil; 8 IPPOCRATE Centro Medico Specialistico, Ladispoli, Rome Italy; 9 Physical Therapy Unit, Primary Health Care Center “El Abajón”, 28231 Las Rozas de Madrid, Spain

**Keywords:** Diseases, Health care, Medical research

## Abstract

Chronic obstructive pulmonary disease (COPD) is a leading cause of morbidity and mortality. Pulmonary rehabilitation (PR) is central to COPD management; however, individuals with musculoskeletal limitations, obesity, or reduced tolerance to land-based rehabilitation (LBR) may benefit from water-based rehabilitation (WBR). To evaluate the comparative effectiveness of WBR, LBR, and control interventions on exercise capacity (EC) and health-related quality of life (HRQoL) in adults with COPD using a systematic review and network meta-analysis (NMA). Randomized controlled trials (RCTs) involving adults with COPD were searched in PubMed, Cochrane Library, PEDro, and Bibliothèque nationale du Luxembourg from inception to July 13, 2025. The primary outcomes were EC, measured using the 6-Minute Walk Test (6MWT), Incremental Shuttle Walk Test (ISWT), and Endurance Shuttle Walk Test (ESWT), and HRQoL, assessed using the Chronic Respiratory Disease Questionnaire (CRDQ) and St. George’s Respiratory Questionnaire (SGRQ). The Risk of bias was assessed using the Risk of Bias 2.0 tool (RoB 2.0). A frequentist NMA was used to estimate the comparative effects and intervention rankings. The certainty of the evidence was rated using the Grading of Recommendations, Assessment, Development, and Evaluations approach (GRADE). Nine RCTs (n = 323) were included in the analysis. WBR was associated with clinically meaningful improvements in EC, particularly in endurance performance (ESWT) and shuttle walking (ISWT), with several effects exceeding the established minimal clinically important difference (MCID) thresholds. HRQoL outcomes were heterogeneous: WBR improved selected CRDQ and SGRQ domains, whereas LBR ranked highest for overall HRQoL. The NMA ranking suggested that WBR had the highest probability of being the most effective intervention for EC and combined EC+HRQoL outcomes, whereas LBR ranked highest for HRQoL alone. Heterogeneity was low for EC, moderate for HRQoL, and high for the combined outcomes. The certainty of evidence ranged from moderate (EC) to low (combined outcomes). WBR is a viable alternative to LBR for improving EC in individuals with COPD and may be particularly beneficial for those with reduced mobility or limited tolerance to land-based training. However, given the limited number of trials and variability across studies, these findings should be interpreted with caution. High-quality and adequately powered RCTs are required to confirm the long-term effects and real-world applicability of these findings.

## Introduction

Chronic Obstructive Pulmonary Disease (COPD) remains the third leading cause of mortality globally and substantially contributes to the burden of chronic respiratory diseases^1^. According to the Global Initiative for Chronic Obstructive Lung Disease (GOLD), COPD is a heterogeneous condition characterized by persistent respiratory symptoms, including dyspnea, cough, sputum production, and exacerbations, arising from airway (bronchitis and bronchiolitis) and/or alveolar (emphysema) abnormalities that cause irreversible airflow limitations^2^.

The two main phenotypes of COPD, emphysema and chronic bronchitis, often coexist and are associated with systemic consequences such as skeletal muscle dysfunction, reduced exercise tolerance, and impaired lung mechanics^3–5^. These manifestations collectively reduce the quality of life (QoL) and functional independence^6,7^. Pulmonary rehabilitation (PR) is the cornerstone of COPD management and has well-established benefits for exercise capacity (EC) and health-related quality of life (HRQoL)^8^. Modern PR emphasizes individualized multidisciplinary programs incorporating exercise training, education, and behavioral support tailored to patient characteristics and comorbidities^9,10^.

However, conventional land-based rehabilitation (LBR) may not be well tolerated by individuals with advanced disease, musculoskeletal limitations, obesity, or marked deconditioning^10^. Water-based rehabilitation (WBR) has emerged as a promising alternative adjunctive modality. Its benefits are derived from the unique physical properties of water—buoyancy, viscosity, and hydrostatic pressure—which allow patients to exercise with reduced joint load, improved movement control, and lower perceived exertion^11–14^.

In addition to musculoskeletal unloading, immersion in water induces specific respiratory and cardiopulmonary adaptations that are particularly relevant for COPD. The hydrostatic pressure exerted on the thorax and abdomen increases expiratory resistance, facilitating more effective exhalation and potentially contributing to a reduction in dynamic hyperinflation and residual volume^7,15^. This mechanism may improve lung mechanics and ventilatory efficiency during exercise. Aquatic exercise has also been associated with reductions in dyspnea perception and fatigue, likely mediated by improved breathing pattern control and a lower ventilatory demand for a given workload^11^. Together, these physiological effects may translate into meaningful improvements in EC, respiratory efficiency, and HRQoL in individuals with COPD, particularly in those who experience limitations or intolerance to conventional LBR.

Buoyancy decreases gravitational stress and facilitates a greater range of motion, whereas water viscosity provides multidirectional resistance that enhances muscular activation and endurance^13,14,16^. Hydrostatic pressure exerts a uniform compressive force on the thorax, increasing the inspiratory workload and potentially strengthening the respiratory muscles^16^. Warm-water immersion (≥ 32 °C) further promotes peripheral circulation and oxygen delivery, supports aerobic performance, and reduces postexercise discomfort^17^.

Although early physiological studies raised concerns about chest wall compression and reduced lung volumes during immersion^15,18–20^, contemporary clinical trials, including the randomized controlled trial by McNamara et al.^11^, have demonstrated that the WBR is safe, feasible, and well tolerated by individuals with COPD. A recent systematic review by Chen et al.^21^ confirmed the beneficial effects of WBR on dyspnea, lung function, and EC, although HRQoL outcomes have only been superficially reported.

Although the beneficial effects of PR on HRQoL in COPD are well established and constitute a core objective of rehabilitation programs^22^, several recent network meta-analyses have evaluated the comparative effects of different exercise-based interventions on EC in individuals with COPD^23,24^. However, these studies primarily focused on land-based or mixed exercise interventions and did not specifically examine WBR within a dedicated framework. In this context, recent randomized trials by McNamara et al.^11^ and Charususin et al.^12^ demonstrated meaningful gains in both EC and HRQoL following WBR, supporting its potential integration into multidisciplinary rehabilitation programs.

Therefore, this systematic review and network meta-analysis aimed to evaluate the comparative effectiveness of WBR in relation to established rehabilitation modalities, including LBR and control conditions, rather than providing an exhaustive synthesis of conventional land-based PR versus control. By focusing on randomized controlled trials (RCTs) in which WBR constituted at least one intervention arm, this study sought to determine whether aquatic-based programs represent a valid and clinically meaningful alternative or complement to LBR in individuals with COPD.

## Methods

This systematic review followed the 2020 Preferred Reporting Items for Systematic Reviews and Meta-Analyses (PRISMA) guidelines^25^. The review protocol was prospectively registered with the Open Science Framework (OSF) (10.17605/OSF.IO/U5E7P).

### Eligibility criteria

The selection of studies was guided by the PICO framework (Population, Intervention, Comparison, Outcome). Eligible studies included RCTs conducted in adults (≥ 18 years) diagnosed with COPD, irrespective of disease severity. The intervention of interest was WBR, compared with either LBR or standard care/control group (CG). Randomized controlled trials comparing LBR with control alone, without a WBR arm, were not included, as their inclusion would have substantially broadened the scope of conventional PR and reduced the clinical coherence of the network meta-analysis focused on aquatic rehabilitation.

The primary outcomes were EC and HRQOL. Validated and reliable outcome measures for EC included the 6-Minute Walk Test (6MWT), Incremental Shuttle Walk Test (ISWT), and Endurance Shuttle Walk Test (ESWT)^26^. The Chronic Respiratory Disease Questionnaire (CRDQ) and St. George’s Respiratory Questionnaire (SGRQ) were used for HRQoL assessment^27–30^. Other disease-specific instruments, such as the Clinical COPD Questionnaire (CCQ) and the COPD Assessment Test (CAT), as well as generic health-related quality-of-life measures (e.g., EQ-5D and SF-36), were not included because they are primarily designed to assess symptom burden or general health status and show lower sensitivity and consistency in detecting rehabilitation-induced changes in COPD populations. Secondary outcomes were predefined. Although some studies reported other physiological or perceptual variables (e.g., SpO₂, dyspnea, respiratory muscle strength, and balance), they were not part of the eligibility criteria and were not included in the quantitative synthesis.

To be included, studies had to report at least one of the specified primary outcomes using the corresponding validated tools. Only RCTs were considered, with no restrictions on publication dates.

### Search strategy

A comprehensive electronic search was conducted on July 13, 2025, in four major databases: PubMed, Cochrane Library, Bibliothèque Nationale de Luxembourg (BnL), and Physiotherapy Evidence Database (PEDro). The search strategy was developed based on the PICO framework and optimized by consulting a research librarian specializing in systematic reviews.

The following specifications were applied:

- Timeframe: From inception to July 13, 2025.
- Language: Articles published in English, French, Spanish, or Portuguese were included.
- Study Design: Only RCTs conducted in adult humans (≥ 18 years) were included.

In addition to the database search, we performed manual backward citation tracking of eligible studies and prior systematic reviews (e.g., Chen et al.^21^) to identify additional relevant trials that were not electronically captured.

The complete PubMed search syntax is detailed below as a representative example, and analogous adaptations were applied to the other databases:

### Study selection

All references retrieved from the database search were imported into a reference manager (Zotero) for automatic and manual duplications. The study selection process was conducted independently by four reviewers (AJZ, AG, PS, and EASR) following PRISMA 2020 guidelines.

During the first screening phase, titles and abstracts were evaluated for relevance to the predefined eligibility criteria. Articles that did not meet the inclusion criteria were excluded from the study. Any disagreements between the reviewers were resolved through discussion.

In the second phase, full-text versions of potentially eligible studies were retrieved and independently assessed by the same four reviewers. Discrepancies regarding study inclusion were resolved by consensus, and a third reviewer (RMA) was consulted when necessary. Three senior team members (FXA, EASR and CC) verified the results of the included studies.

To be eligible, studies had to:

- Be RCTs,
- Involve adults ( ≥ 18 years) with a clinical diagnosis of COPD,
- Comparison of a WBR intervention with either LBR or a CG
- At least one of the primary outcomes, EC or HRQoL, was assessed using validated pre-specified tools (6MWT, ISWT, ESWT, CRDQ, and SGRQ).

The OSF-registered protocol (June 2023) was updated in early 2025 to extend the search strategy to July 2025 and include a planned network meta-analysis.

### Risk of bias assessment

The methodological quality of the included studies was assessed using the Cochrane Risk of Bias 2.0 (RoB 2) tool for randomized controlled trials^31^. This assessment was independently performed by four reviewers (AJZ, AG, EASR, and PS) in five domains.

1. Bias arising from the randomization process;
2. Bias due to deviations from intended interventions;
3. Bias due to missing outcome data;
4. Bias in the measurement of the outcome;
5. Bias in the selection of the reported result.

Each domain was rated as “low risk,” “some concerns,” or “high risk” according to the Cochrane guidelines. The overall risk of bias judgement for each study followed the following criteria:

- “Low risk” if all domains were rated as low risk.
- “Some concerns” if at least one domain raised concerns but none were high-risk;
- “High risk” if one or more domains were rated as high risk, or if multiple domains raised concerns.

Discrepancies in domain-level or overall ratings were resolved through discussions among the assessors until a consensus was reached. The final agreement was documented for each study.

### Effect measures

For continuous outcomes, the treatment effect was summarized using either the mean difference (MD) or standardized mean difference (SMD) with 95% confidence intervals (CI), depending on the consistency of the measurement scales across studies. Specifically:

- If the same instrument and scale were used across the studies (e.g., the 6-Minute Walk Test in meters), the MD was applied.
- If different instruments or scales were used to assess the same construct (e.g., HRQoL via the CRDQ and SGRQ), the SMD was employed to allow comparability.

When studies reported only change-from-baseline scores or post-intervention values, the data were extracted accordingly. When the standard deviations (SDs) of the change scores were not available, they were imputed using established methods, including estimation via correlation coefficients, as recommended in the Cochrane Handbook for Systematic Reviews of Interventions (Section 6.5.2.8)^32^.

The primary outcomes were:

- EC was measured using the 6MWT, Incremental Shuttle Walk Test (ISWT), and Endurance Shuttle Walk Test (ESWT).
- HRQoL was assessed using the Chronic Respiratory Disease Questionnaire (CRDQ) and St. George’s Respiratory Questionnaire (SGRQ).

### Data collection and synthesis methods

Data extraction was performed independently by two reviewers (AJZ and AG) using a pre-defined and piloted data collection form. All extracted data were verified by two additional reviewers (EASR and PS) to ensure accuracy and consistency. Discrepancies were resolved through discussion and consensus.

Data Harmonization Procedures Before conducting quantitative analyses, all extracted outcome data underwent a multistep harmonization and verification process. First, the two reviewers independently extracted the means, standard deviations (SDs), sample sizes, and measurement scales for EC and HRQoL. A second pair of reviewers independently verified the accuracy and internal consistency of all extracted data. When studies reported only change-from-baseline values or post-intervention scores, the data were transformed following the recommendations of the Cochrane Handbook for Systematic Reviews of Interventions (Section 6.5.2.8)^32^.

If SDs for change scores were not reported, they were imputed using established formulas based on the correlation coefficients derived from similar COPD exercise trials. All outcome values were harmonized to ensure consistency across units and scoring directions (e.g., reversing the SGRQ scores when necessary), thus enabling comparability across studies. Only after this harmonization process was complete, pairwise meta-analysis and network meta-analysis (NMA) were conducted.

For each included study, the following data were extracted:

- Study characteristics: authors, year of publication, country, study design.
- Participant characteristics: sample size per group, mean age, sex distribution, COPD severity, and diagnostic criteria. Although participant characteristics, such as mean age, sex distribution, COPD severity, and diagnostic criteria, were extracted when available, these variables were inconsistently reported across trials. To avoid presenting incomplete or heterogeneous information, only the characteristics consistently reported by all studies (e.g., sample size and intervention details) are included in Table 1.
- Intervention details: type of intervention (WBR, LBR, or CG), exercise modalities, session duration and frequency, total program duration, and pool temperature (if applicable).
- Outcome data: Mean and standard deviation (SD) at baseline and post-intervention (or change scores) were extracted for the primary outcomes: EC (6MWT, ISWT, and ESWT) and HRQoL (CRDQ and SGRQ). Although some studies reported additional variables (e.g., dyspnea scores, SpO₂, respiratory muscle strength, and balance), these were not pre-specified as eligibility criteria and were therefore not included as secondary outcomes in the quantitative synthesis. Only pre-defined primary outcomes were used to determine study inclusion and conduct analyses.

**Table 1:** Characteristics of the included studies.

Author, Year	Country	Sample Size / COPD Severity	Comparison	Primary Outcomes	Core Intervention Characteristics (FITT)	Description of Control	Main Findings (between-group)
**McNamara et al.**, ^11^	Australia	n = 53; GOLD II–III; mean age 72 ± 9 y; 43% male	WB vs LB vs CG	6MWT, ISWT, ESWT, CRDQ	12 weeks; 3×/week; 60 min; heated pool 32–33 °C; supervised	Usual care	WB > CG (6MWT, ISWT, ESWT, CRDQ-fatigue; all p < 0.05); WB > LB (ISWT, ESWT; p < 0.05).
**Charususin et al.**, ^12^	Thailand	n = 14; moderate COPD (GOLD not reported); mean age 66–69 y; sex 2/5 F/M in both groups	WB vs LB (supervised vs unsupervised)	6MWT, ISWT, ESWT	4 weeks; 3×/week; supervised aquatic vs unsupervised home LB	Home-based unsupervised	WB > LB for ESWT (p < 0.05); no differences for 6MWT or ISWT.
**Özdemir et al.**, ^41^	Turkey	n = 50; moderate–severe COPD; all male; mean age WB 60.9 ± 8.8 / CG 64.1 ± 8.9 y	WB vs CG	6MWT, CRDQ	8 weeks; 3×/week; 60 min; pool temperature NR	No exercise	WB > CG for 6MWT and CRDQ-total (both p < 0.05).
**de Souto Araujo et al.**, ^42^	Brazil	n = 32; GOLD II–IV; mean ages: CG 71.1 ± 10.1 y; FG 56.9 ± 7.9 y; AG 62.4 ± 9.9 y; sex: CG 3/8 F/M, FG 5/8 F/M, AG 4/4 F/M	WB vs LB vs CG	6MWT, SGRQ	12 weeks; 2×/week; low-intensity aquatic exercise	No exercise	LB > CG for SGRQ (p < 0.05); no significant between-group differences for 6MWT
**Felcar et al.**, ^43^	Brazil	n = 36; GOLD II–IV; mean age 68–69 y; 55% male	WB vs LB	6MWT, ISWT, CRDQ	8 weeks; 2×/week; 60 min; pool 32–33 °C	—	Both groups improved 6MWT, ISWT, CRDQ (p < 0.05); no significant WB vs LB differences.
**de Castro et al.**, ^44^	Brazil	n = 31; moderate COPD; mean age 64–65 y; 53% male	WB vs LB	6MWT, ISWT	12 weeks; 2×/week; 60 min; supervised	—	LB > WB for 6MWT and ISWT (p < 0.05).
**Liu et al.**, ^46^	China	n = 45; GOLD I–IV (mainly II–III); mean age 65–66 y; 60–80% male	WB vs LB vs CG	6MWT, SGRQ	12 weeks; 2×/week; WB pool 26–30 °C; Liuzijue-based protocol	No exercise	WB > CG (6MWT + 90.5 m; SGRQ p < 0.05); LB also improved vs CG.
**Wadell et al.**, ^47^	Sweden	n = 43; GOLD II–III; mean age ~64 ± 6 y; 37% male	WB vs LB vs CG	ISWT, ESWT, SGRQ	12 weeks; 3×/week; 45 min; high-intensity (80–90% HRpeak); pool 33–34 °C	No exercise	WB > CG for ESWT ( + 179 m); LB > CG for ISWT ( + 25 m); CG worsened in SGRQ.
**Gallo-Silva et al.**, ^45^	Brazil	n = 19; GOLD I–IV; mean age UCG 66.5 ± 9.5 y / TG 66.3 ± 6.5 y; all male	WB vs CG	SGRQ, 6MWT	24 sessions; 3×/week; 60 min; pool 32 °C; interval aerobic	Usual care	WB > CG for SGRQ domains (p < 0.05); 6MWD + 74.9 m (p < 0.05).

*WB* water-based, *LB* land-based, *CG* control group, *F/M* female/male, *6MWT* 6-Minute Walk Test, *6MWD* 6 min walk distance, *ISWT* incremental shuttle walk test, *ESWT* endurance shuttle walk test, *CRDQ* chronic respiratory disease questionnaire, *SGRQ* St. George’s respiratory questionnaire, *GOLD* global initiative for obstructive lung disease, *FITT* frequency, intensity, time, type, *HRpeak* peak heart rate.

When outcome data were incomplete or reported in a non-standard format, estimations were performed based on the guidance from the Cochrane Handbook for Systematic Reviews of Interventions, including the use of correlation coefficients to estimate missing standard deviations (SDs) for change scores^32^. The authors were contacted to obtain missing numerical data when necessary.

A qualitative synthesis of the included randomized controlled trials was conducted, and the key characteristics of each study are summarized in Table 1. This table includes the study design, participant details, interventions, outcome measures, and main findings relevant to EC and HRQoL. The full electronic search strategies for all databases are provided in Supplementary Material.

A network meta-analysis (NMA) was conducted after data harmonization. The interventions were categorized into three nodes: WBR, LBR, and CG. The quantitative synthesis included both direct and indirect comparisons using a frequentist NMA framework, as detailed in Section 2.8 (Statistical).

### Certainty of evidence (GRADE)

The Grading of Recommendations Assessment, Development, and Evaluation (GRADE) approach was used to assess the certainty of the evidence for the main comparisons and outcomes once the network meta-analysis was complete^33^. The primary outcomes considered for the GRADE assessment included EC (6MWT, ISWT, and ESWT) and HRQoL (CRDQ and SGRQ) measurements.

The GRADE methodology considers five domains:

1. Risk of bias, based on RoB 2.0 evaluations;
2. Inconsistency, assessed through heterogeneity and coherence across studies;
3. Indirectness was evaluated based on the relevance of the population, intervention, comparator, and outcome.
4. Imprecision, based on confidence intervals and sample size;
5. Publication bias was assessed using funnel plots and statistical tests if data were allowed.

### Statistical analysis

For statistical analysis, R Ver. 4.1.3 (R Foundation for Statistical Computing, Institute for Statistics and Mathematics, Welthandelsplatz 1, 1020 Vienna, Austria) and the metafor package^34^ were used for the analysis.

We chose a network meta-analysis (NMA) framework because the included RCTs formed a connected network of comparisons between the three main intervention nodes: WBR, LBR, and CG. Several trials directly compared WBR and LBR, whereas others compared either WBR or LBR with CG; however, direct WBR–CG comparisons were scarce. Therefore, a traditional pairwise meta-analysis limited to direct contrast would underutilize the available evidence. The NMA approach allowed us to synthesize both direct and indirect comparisons across all three nodes, increase the statistical power, and derive a coherent ranking of interventions for EC, HRQoL, and combined outcomes.

A multivariate frequentist network meta-analysis (NMA) was performed to evaluate the effects of the three interventions (water-based, land-based, and control) on three outcome variables: EC, assessed using the 6MWT, incremental shuttle test, and endurance shuttle test; HRQoL, assessed using the Chronic Respiratory Disease Questionnaire and St. George’s Respiratory Questionnaire scales; and the combination of both scales, assessed using the 6MWT and St. George’s Respiratory Questionnaire. The standardized mean difference (SMD) between groups and correlations between the different scales reported in the literature were used^35–39^. The calculated effect sizes were defined as small ( < 0.2), moderate (0.2–0.8), and large ( > 0.8). The St. George’s Respiratory Questionnaire was the only inverse variable; therefore, its scoring was reversed using appropriate formulas^40^. In addition to the separate analyses of EC and HRQoL, a combined EC+HRQoL outcome was explored to provide an integrated assessment of the multidimensional rehabilitation benefits. This combined analysis was considered exploratory and complementary and was not intended to replace the interpretation of individual outcomes.

The adequacy of the fixed- or random-effects model was determined using a likelihood ratio test (LRT) between both, evaluating the level of significance and the values of the Akaike Information Criterion (AIC), in which the lowest value was the best.

The transitivity assumption was assessed by assuming that effect modifiers were similarly distributed across studies. To this end, the model was controlled by adjusting for age, Body Mass Index, and female/male ratio as confounding variables to determine whether they significantly influenced the NMA results and their impact on the level of heterogeneity.

Heterogeneity and model consistency were assessed by analyzing the LRT and AIC between the models with and without covariates and between the consistent and inconsistent models, respectively. The contribution of each study to heterogeneity was assessed using a contribution matrix for each direct comparison.

Between-study variance was calculated using τ2 with the Restricted Maximum Likelihood (REML) estimator, Cochrane Q test, and I^2^ estimator. The latter was defined as follows: 0–30%, unimportant heterogeneity; 30–50%: moderate heterogeneity; 50–75%: high heterogeneity; 75–100%: significant heterogeneity; and regression coefficient (R^2^) of the model.

The effectiveness of the treatments was analyzed using P-scores and significance level analysis for pairwise comparisons.

Finally, publication bias was analyzed using a funnel plot adjusted for each comparison and Egger’s test.

## Results

### Study selection

A total of 238 records were identified through a database search (PubMed, Cochrane Library, PEDro, and Bibliothèque nationale du Luxembourg) from database inception to July 2025, and no additional records were identified through manual reference checking. After removing 12 duplicates, 226 records were subjected to title and abstract screening, of which 214 were excluded because they did not meet the eligibility criteria.

Twelve full-text articles were assessed for eligibility. Following a detailed review, three articles were excluded for the following reasons:

1. No prespecified primary outcomes (EC or HRQoL) were reported.
2. Secondary publication providing duplicate data from a previously included RCT;
3. Insufficient outcome reporting, preventing extraction of eligible quantitative data.

Ultimately, nine RCTs^11,12,41–47^ fulfilled all the inclusion criteria and were included in both the qualitative synthesis and network meta-analysis.

The review process followed the original OSF-registered protocol (June 2023). An update was implemented in early 2025 to extend the search period to July 2025 and incorporate the planned network meta-analysis while maintaining the same prespecified eligibility criteria.

**Fig. 1:**
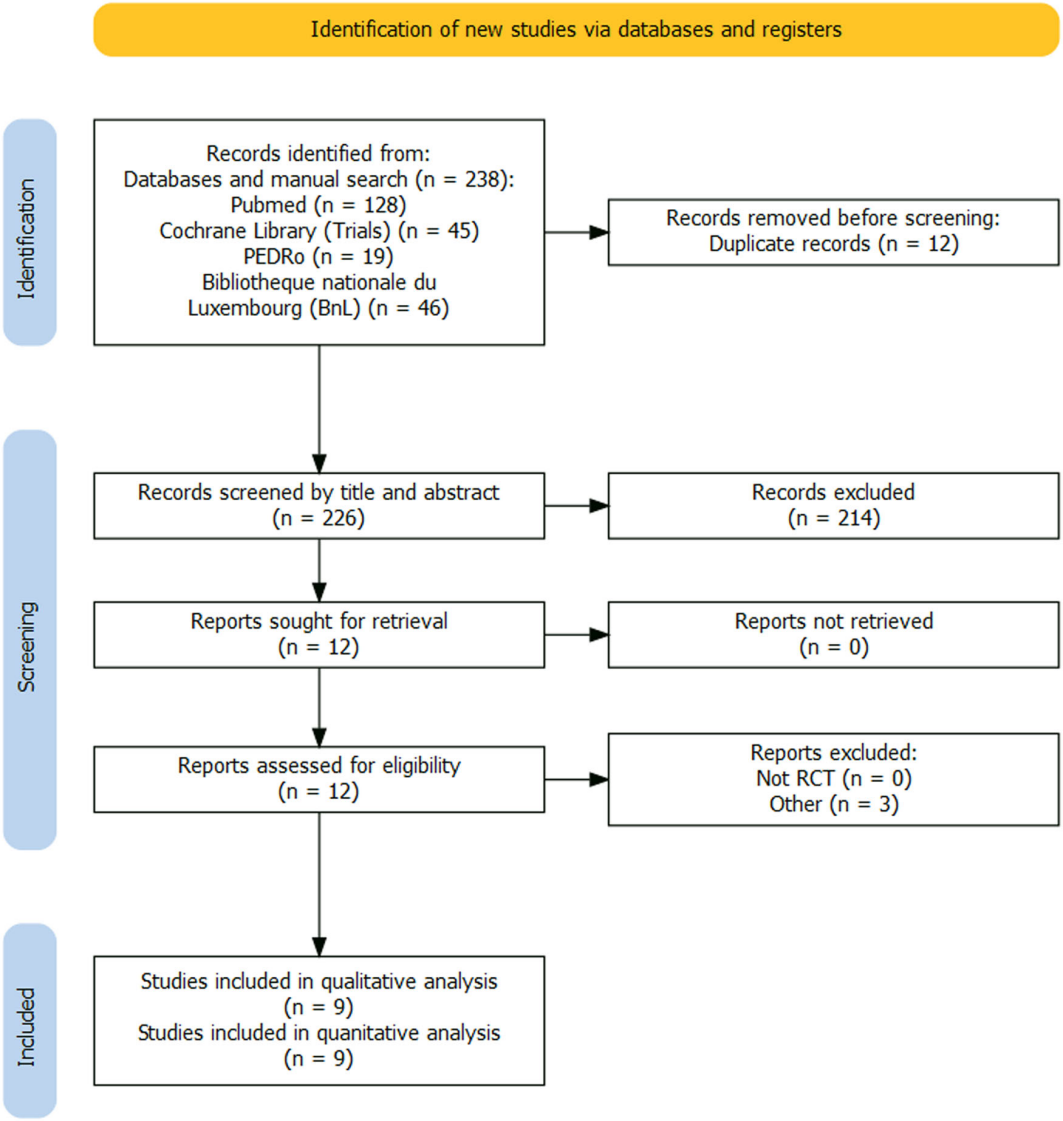
PRISMA 2020 flow diagram of the study selection process. The diagram illustrates the identification, screening, eligibility, and inclusion of studies considered for the systematic review and network meta-analysis.

The complete study selection workflow, including identification, screening, eligibility assessment, and reasons for exclusion, is presented in Fig. 1 (PRISMA 2020 flow diagram).

To improve interpretability, detailed descriptions of individual trials were minimized in the narrative text and are primarily presented in tables, whereas the main text emphasizes the comparative effectiveness across interventions and outcomes.

### Study characteristics

The nine randomized controlled trials included in this review, published between 2004 and 2021, enrolled 323 adults with COPD. Across studies, individuals were typically older adults, with mean ages ranging from 60–72 years. Most patients were predominantly male, with male proportions generally between 50 and 80%, reflecting the demographic patterns that are commonly observed in COPD cohorts.

The severity of COPD across the trials was mainly within the moderate-to-severe range (GOLD II–III). Several studies have also included individuals with mild or very severe disease (GOLD I and IV), resulting in heterogeneous but predominantly moderate airflow limitation.

These studies were conducted in Australia^11^, Thailand^12^, Turkey^41^, Brazil^42–45^ China^46^, and Sweden^47^. All trials compared the WBR to either the LBR or CG. Interventions were performed in heated pools, most commonly at 32–34 °C, and involved aerobic, endurance, and resistance training adapted to the functional limitations of individuals with COPD. The training duration ranged from 4–24 weeks, with a frequency of 2–3 supervised sessions per week. The control group received regular or no exercise educational guidance. Most interventions were delivered through supervised sessions, although the levels of supervision and professional backgrounds have not been consistently reported.

EC was assessed using validated tests: the 6MWT, Incremental Shuttle Walk Test (ISWT), and Endurance Shuttle Walk Test (ESWT), whereas HRQoL was evaluated using the Chronic Respiratory Disease Questionnaire (CRDQ) or St. George’s Respiratory Questionnaire (SGRQ). All included trials provided pre- and post-intervention measurements for at least one primary outcome.

A complete summary of the population characteristics, intervention parameters, and between-group findings is presented in Table 1.

### Risk of bias in studies

#### Randomization process (selection bias)

For Domain 1 (bias arising from the randomization process), most trials were judged to be at low risk, as they adequately described sequence generation and presented balanced baseline characteristics. Three studies were rated as concerning this domain: Wadell et al.^47^, de Souto Araujo et al.^42^, and Özdemir et al.^41^, primarily because of insufficient reporting on the randomization procedure and allocation concealment, which limited the ability to confirm whether randomization was implemented appropriately. Although these studies showed minor baseline imbalances, they were not substantial enough to classify them as high-risk. Overall, this domain showed no studies at high risk, three with some concerns, and the remainder at a low risk.

#### Deviations from intended interventions (performance bias)

In Domain 2, most trials were judged to be concerning, primarily because adherence to the intervention protocol, supervision level, and monitoring of co-interventions were insufficiently reported. Only Liu et al.^46^ and Charususin et al.^12^ provided adequate information regarding intervention fidelity and supervision and were therefore rated as having low risk in this domain. All remaining studies lacked detailed reporting on adherence, protocol deviations, or co-interventions, resulting in a classification of some concerns.

#### Missing outcome data (attrition bias)

For Domain 3, four studies—McNamara et al.^11^, Charususin et al.^12^, Liu et al.^46^, and Wadell et al.^47^—were judged to be at low risk, as they provided complete follow-up data or used appropriate methods to handle missing outcomes. In contrast, Özdemir et al.^41^, de Souto Araujo et al.^42^, and Felcar et al.^43^ were rated as high risk because of substantial losses to follow-up or insufficient reporting that could plausibly bias outcome estimates. Finally, Gallo-Silva et al.^45^ and de Castro et al.^44^ were classified as having some concerns, primarily because incomplete outcome reporting or unclear handling of missing data introduced uncertainty but did not indicate a high risk of bias.

#### Measurement of the outcome (detection bias)

For Domain 4, all included studies were judged to have a low risk of bias. Outcome measures, such as the 6MWT, ISWT, ESWT, CRDQ, and SGRQ, were administered using standardized and validated protocols, and no evidence was identified to suggest systematic differences in outcome assessment across the intervention groups. Consequently, this domain did not raise any methodological concerns in any of the trials.

#### Selection of the reported result (reporting bias)

For Domain 5, four studies were assessed as having *low risk* of bias, specifically Özdemir et al.^41^, Felcar et al.^43^, Gallo-Silva et al.^45^, and Liu et al.^46^, all of which reported outcomes transparently and consistently with their prespecified protocols. One study, Charususin et al.^12^, was judged to be at *high risk* owing to selective reporting and discrepancies between the prespecified and reported outcomes. The remaining studies—Wadell et al.^47^, de Souto Araujo et al.^42^, McNamara et al.^11^, and de Castro et al.^44^—were categorized as having *some concerns*, largely because of incomplete reporting, multiple eligible outcome measurements, or insufficient information regarding prespecification of analyses.

#### Overall risk of bias

Overall, three studies, Özdemir et al.^41^, de Souto Araujo et al.^42^, and Charususin et al.^12^, were judged to have a *high risk* of bias, primarily due to substantial missing outcome data, insufficient methodological reporting, or selective outcome reporting. Four studies—Wadell et al.^47^, McNamara et al.^11^, Felcar et al.^43^, and de Castro et al.^44^—were categorized as presenting *some concerns*, generally related to incomplete reporting or uncertainties across one or more RoB 2.0 domains. Only two studies, Gallo-Silva et al.^45^ and Liu et al.^46^, demonstrated a *low overall risk* of bias, reflecting adequate methodological rigor and consistent reporting across all domains.

**Fig. 2:**
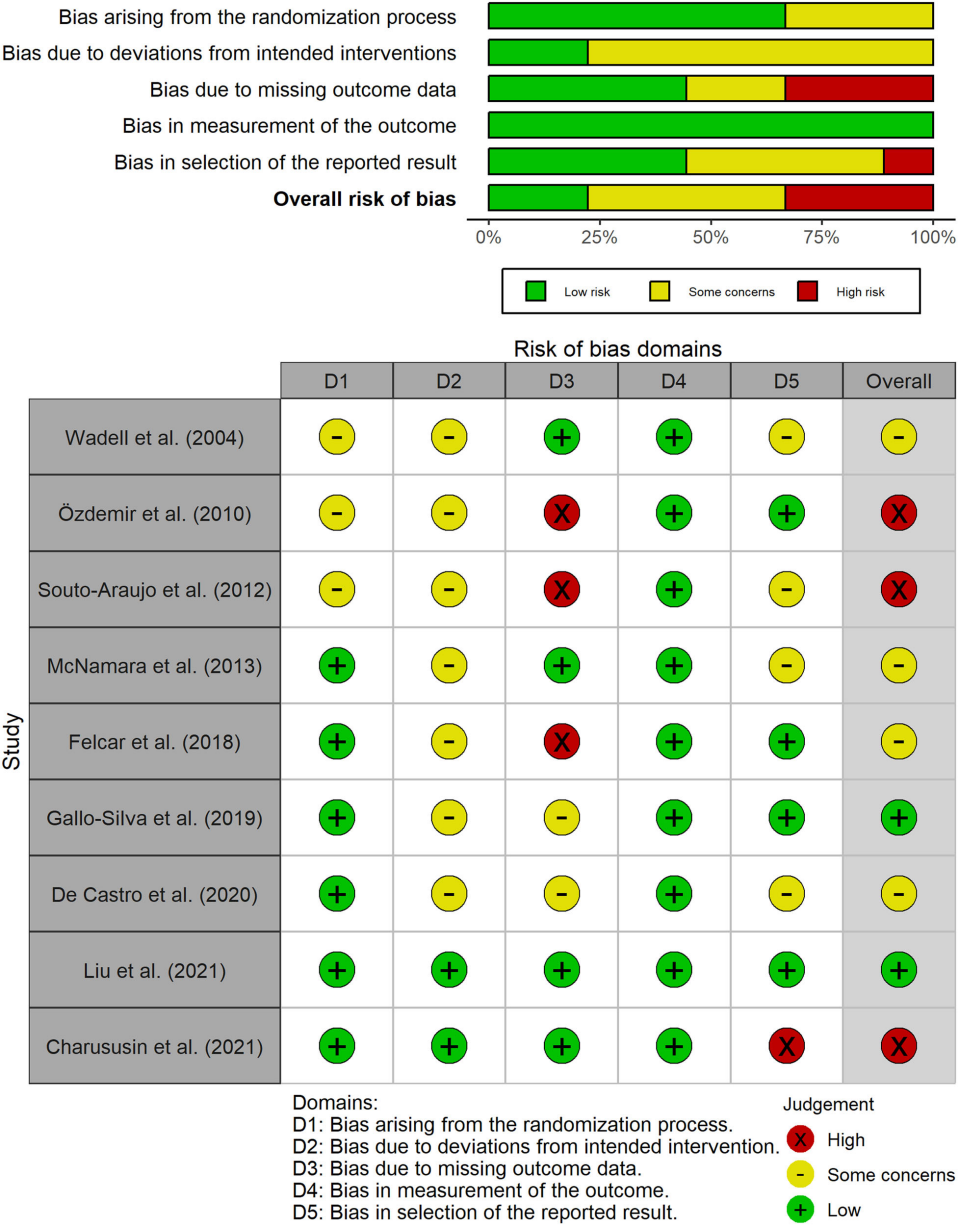
Risk of bias assessment of included randomized controlled trials using the RoB 2.0 tool. Green indicates low risk of bias; yellow indicates some concerns; red indicates high risk of bias.

A visual summary of the domain-level assessments is presented in Fig. 2 (RoB 2.0, traffic light visualization).

### Effects of water-based rehabilitation on exercise capacity

The results are presented as comparative and pooled estimates derived from the network meta-analysis, focusing on between-intervention effects and clinical relevance rather than individual trial-level descriptions. EC was assessed in the included studies using the 6MWT, incremental shuttle Walk Test (ISWT), and Endurance Shuttle Walk Test (ESWT). The minimal clinically important differences (MCID) established for chronic respiratory disease were used to evaluate the clinical relevance of changes, following the measurement properties summarized in the ERS/ATS official systematic review by Singh et al.^48^. Specifically, MCIDs of 25–33 m for the 6MWT^48,49^, 35–36 m for the ISWT^50^, and 156 s (or approximately 188 m) for the ESWT^51^ were applied. Statistical significance was determined based on the reported p-values and confidence intervals.

Özdemir et al.^41^ evaluated a 4-week WBR program in 50 men with moderate-to-severe COPD. No statistically significant improvement was observed in the 6MWT within the WBR group (425.0 ± 44.3–431.1 ± 61.4 m; p = 0.557), whereas the control group showed a significant decline (383.9 ± 98.6–344.7 ± 107.8 m; p = 0.004). Although the between-group difference at one month favored WBR, the change did not reach an MCID of 25–33 m and was mainly driven by deterioration in the control group rather than true improvement with aquatic exercise alone. No additional EC outcomes (ISWT or ESWT) were observed. The authors observed improvements in the CRDQ domains and anxiety levels within the WBR group; however, the short intervention duration and incomplete reporting of adherence introduced uncertainty regarding the robustness of these results.

De Souto Araujo et al.^42^ examined an 8-week low-intensity aquatic training program in individuals with moderate-to-severe COPD. After accounting for dropouts (final n = 32), only the aquatic group achieved a statistically significant and clinically meaningful improvement in the 6MWT (434.6 ± 120.9 → 490.9 ± 137.8 m; p = 0.02), surpassing the MCID of 25–33 m^48,49^. Neither the land-based nor the control group demonstrated significant gains, and the control group showed functional decline. The aquatic group also showed improved dyspnea scores and BODE index, while changes in SGRQ domains, although favorable, did not reach statistical significance. Overall, this study indicates that WBR may provide greater functional benefits than land-based training in individuals with moderate-to-severe COPD.

McNamara et al.^11^ evaluated EC using the 6MWT, ISWT, and ESWT after eight weeks of supervised training in individuals with COPD and physical comorbidities. The WBR group improved the 6MWT from 349 ± 91–397 ± 68 m (Δ +48 m; p < 0.001), whereas the LBR group improved from 300 ± 142–343 ± 131 m (Δ +43 m; p = 0.001), both of which exceeded the updated MCID threshold of 25–33 m. WBR demonstrated significantly greater gains than LBR in the ISWT (186 ± 93–235 ± 96 m; Δ +49 m; p = 0.001), surpassing the updated MCID of 35–36 m^50^, and in the ESWT (271 ± 153–591 ± 367 m; Δ +321 m; p = 0.004), exceeding the MCID threshold of 156 s or 188 m^51^. The WBR also produced superior improvements in the CRDQ-fatigue compared with the LBR and control groups. Overall, this study demonstrates that WBR provides clinically and statistically superior improvements in EC, particularly endurance, compared to LBR in individuals with COPD.

Felcar et al.^43^ compared two equivalent six-month high-intensity training protocols in individuals with COPD undergoing either water- or land-based exercises. After 60 supervised sessions, both groups demonstrated significant improvements in the 6MWT (land-based: from 469 ± 77–524 ± 81 m, and water-based: from 478 ± 77–519 ± 93 m; both p < 0.001), as well as in ISWT performance, maximal and submaximal EC, and peripheral and respiratory muscle strength. Both the 6MWT changes of 55 m (land) and 41 m (water) exceeded the updated MCID threshold of 25–33 m, indicating clinically meaningful improvements in both groups^49^. Consistent with the authors’ interpretation, effect sizes favored the water-based group for several outcomes, suggesting potentially greater functional responsiveness. Importantly, no significant between-group differences were found for any EC outcome, indicating that the two environments produced comparable benefits for individuals with COPD in terms of EC.

De Castro et al.^44^ evaluated a 3-month high-intensity training program in individuals with COPD allocated to land-based (LG) or water-based (WG) exercise groups. Both groups showed improved EC, but only the LG showed statistically significant gains in the 6MWT (from 487 ± 56–532 ± 71 m; p < 0.001) and ISWT (479 ± 143–531 ± 130 m; p = 0.04). The ISWT improvement exceeded the updated MCID of 35–36 m^50^, whereas the 6MWT gain did not exceed the MCID threshold of 25–33 m^48,49^. In contrast, WG did not demonstrate significant changes in either walking test. These results indicate that in this trial, land-based high-intensity training elicited more pronounced improvements in functional EC than water-based training did.

Liu et al.^46^ evaluated the effects of water-and land-based Liuzijue exercises over 12 weeks in individuals with COPD. Both the WBR and LBR groups showed significantly improved 6MWT performance; however, the magnitude of the change differed substantially. The WBR group increased their 6 MWD by 90.5 m (395.6 ± 63.9–486.1 ± 78.5 m; p < 0.001), clearly surpassing the updated MCID of 25–33 m^48,49^. The LBR group improved by 18.8 m (432.2 ± 65.9–451.0 ± 62.1 m; p = 0.03), a statistically significant but not clinically meaningful change^49^. Only the WBR group showed a significantly greater 6MWT improvement than the control group (p = 0.03). These findings suggest that water-based Liuzijue provides clinically relevant gains in functional EC that exceed those achieved through land-based training.

Wadell et al.^47^ compared the WBR, LBR, and control groups over 12 weeks using the ISWT and ESWT. The WBR group demonstrated a statistically and clinically significant improvement in ESWT performance (+ 179 m), clearly surpassing the ESWT MCID of 156 s/188 m^51^. The LBR group showed improved ISWT by +25 m, but this change fell below the updated MCID threshold of 35–36 m^50^. No significant improvements were observed in the control group, which also showed a deterioration in the SGRQ scores. These findings support the superior effect of water-based training on endurance.

Gallo-Silva et al.^45^ evaluated the effects of a water-based aerobic interval training program in 19 individuals with COPD allocated to a training group (TG) or a usual-care CG. Functional capacity was assessed using only the 6MWT. After 24 sessions, the TG showed a statistically significant improvement in the 6MWT distance (from 466.4 ± 76.0–541.3 ± 66.7 m; p < 0.05), an increase of 75 m that clearly exceeds the established MCID of 25–33 m for the 6MWT, whereas no significant change occurred in the usual-care CG (439.8 ± 102.4–442.2 ± 93.7 m). The between-group differences favored water-based interventions, with a large effect size ( > 0.8). These results indicate that water-based aerobic interval training significantly improves EC in individuals with COPD.

In a recent pilot RCT, Charususin et al.^12^ randomized 14 individuals with COPD to an 8-week, twice-weekly, supervised water-based exercise program or an unsupervised home-based land exercise program. The baseline walking performance (6MWT, ISWT, and ESWT) was comparable between the groups. After training, the water-based group achieved a very large gain in endurance capacity, with ESWT increasing from 352.3 ± 245.5–985.7 ± 304.7 s, whereas the land-based group improved only from 570.0 ± 269.4–615.4 ± 281.0 s; the adjusted between-group difference was 548.8 s (95% CI 274.5–823.1; p = 0.001), clearly exceeding the established MCID for ESWT in COPD^48,51^. In contrast, no significant between-group differences were observed for the 6MWT or ISWT (both p > 0.05), indicating that, in this small sample, water-based training specifically enhanced endurance EC rather than general walking performance.

Overall, most studies reported improvements in EC in favor of WBR, with several trials demonstrating changes that exceeded both statistical significance and the established MCID thresholds. While gains in general walking performance (6MWT and ISWT) varied across studies, water-based interventions consistently produced clinically meaningful enhancements in endurance performance (ESWT). These findings support WBR as a beneficial rehabilitation modality associated with statistically significant improvements in EC compared with the control, although the interpretation of clinical magnitude should consider heterogeneity and established MCID thresholds.

### Effects of water-based rehabilitation on quality of life

Quality-of-life outcomes were synthesized across studies using pooled and comparative estimates from the network meta-analysis, with individual trial data summarized in tables to enhance clarity and interpretability. Seven RCTs assessed the impact of WBR on HRQoL using the Chronic Respiratory Disease Questionnaire (CRDQ) or St. George’s Respiratory Questionnaire (SGRQ)^11,41–43,45–47^. The MCID was defined as ≥4 points for the SGRQ total score^52^ and ≥0.5 points per item for the CRDQ, reflecting clinically meaningful changes across its domains^53^.

Özdemir et al.^41^ evaluated the effects of a four-week water-based PR program on HRQoL in men with COPD using the CRDQ. The authors reported statistically significant improvements (p < 0.05) in dyspnea, emotional function, mastery, and total CRDQ scores in the water-based group. However, the numerical domain values were not provided, which prevented the determination of whether these changes met the established MCID thresholds.

De Souto Araujo et al.^42^ assessed HRQoL using the SGRQ in three groups: the WBR, LBR, and CG. After eight weeks, the LBR group demonstrated statistically significant improvements in all SGRQ domains (p < 0.05), reflected by reductions exceeding the 4-point MCID for clinically meaningful changes^52^. The WBR group showed small decreases ( > 4 points) across all domains, but these did not reach statistical significance, indicating limited HRQoL despite improvements in other clinical outcomes. Conversely, the CG experienced deterioration in SGRQ scores. Overall, only the LBR group achieved clear and clinically important gains in HRQoL, whereas WBR exercise produced modest but non-significant improvements.

In a randomized trial by McNamara et al.^11^, individuals undergoing WBR demonstrated significantly greater improvements in Chronic Respiratory Disease Questionnaire (CRDQ) scores than those in the control group, particularly in the dyspnea, fatigue, and emotion domains (all p < 0.05). Notably, the fatigue domain improved by 4.7 points compared with the control group (p = 0.009), exceeding the established MCID of 0.5 points per item^53^, thus confirming the clinical relevance of this change. Although WBR also outperformed LBR in the fatigue domain (between-group difference: +3.1 points, p = 0.009), no statistically significant differences were observed between WBR and LBR in the remaining CRDQ domains, indicating comparable overall HRQoL benefits between the two interventions.

Felcar et al.^43^ evaluated CRDQ outcomes at three and six months following a 6-month high-intensity training program delivered either in water or on land. Both groups demonstrated statistically significant improvements in all CRDQ domains at six months (p < 0.05), with no significant between-group differences. The effect size values reported by the authors indicated moderate-to-large clinical effects in both groups at 6 months (water: 1.43; land: 1.04), consistent with the established benchmarks for clinically meaningful changes^54^. These findings suggest that prolonged high-intensity training yields meaningful improvements in HRQoL, regardless of the training environment.

Gallo-Silva et al.^45^ evaluated the effects of a 24-session water-based aerobic interval training program on HRQoL in individuals with COPD using St. George’s Respiratory Questionnaire (SGRQ). The training group showed statistically significant improvements across all SGRQ domains (Symptoms, Activities, Impact, and Total score), whereas the control group showed deterioration over time (all p < 0.05). Although absolute post-intervention domain values were not provided, the reported between-group differences clearly favored the intervention group. Based on the direction and magnitude of these changes, the improvements likely exceeded the established 4-point MCID threshold for clinically meaningful changes in the SGRQ^52^. Overall, this study provides supportive evidence that water-based aerobic training enhances HRQoL in individuals with COPD through statistically significant and clinically relevant improvement.

Liu et al.^46^ reported significant improvements in all SGRQ domains following a 12-week water-based Liuzijue program in individuals with COPD. Compared with the control group, the water-based group showed statistically significant reductions in symptoms (p = 0.005), impact (p = 0.018), and total SGRQ score (p = 0.036), all of which exceeded the established 4-point MCID threshold for clinically meaningful change^52^. No significant differences were observed between the water- and land-based groups, although both interventions demonstrated superior HRQoL improvements compared with the control group.

Wadell et al.^47^ compared high-intensity group training in water versus land over 12 weeks and evaluated HRQoL using the SGRQ and SF-36. The control group showed a significant deterioration in the total SGRQ score ( + 5.3), whereas both training groups maintained stable overall scores. The water-based group demonstrated a significant improvement in the SGRQ activity domain ( − 5.1 units; p = 0.009), exceeding the established 4-point MCID threshold^52^. Additionally, the water group exhibited a clinically meaningful improvement in the SF-36 physical component score (from 33–39; p = 0.015), which was greater than the reported MCID benchmarks in populations with chronic disease. In contrast, no significant improvement in the HRQoL was observed in the land-based group. These results indicate that high-intensity aquatic training may confer additional benefits for perceived physical health and activity-related HRQoL compared with land-based programs while preventing the decline observed in the control group.

### Network meta-analysis results

#### Transitivity

The likelihood ratio test (LRT) indicated that age significantly influenced EC outcomes, whereas age and body mass index (BMI) significantly influenced HRQoL outcomes. No moderator demonstrated a significant effect on the combined EC and quality-of-life outcomes. These findings confirm that effect modification must be accounted for in adjusted models (Supplementary Material, Table 1).

#### Heterogeneity and consistency

In the heterogeneity assessment, the random-effects model showed a better fit than the fixed-effects model for all outcomes except for EC, where the reduced fixed-effects model provided the best fit (lower AIC and non-significant LRT).

In consistency testing, the consistent model outperformed the inconsistent full model across all domains (nonsignificant LRT; lower AIC), indicating that the assumption of consistency was satisfied (Supplementary Table 2).

For EC, the models adjusted for BMI, sex ratio, and age were not significant (p = 0.312, p = 0.310, and p = 0.117, respectively), and the unadjusted reduced model demonstrated a lower AIC (84.524 vs. 85.501; 84.524 vs. 85.495; 84.072 vs. 84.524).

The pairwise comparisons contributing most strongly to heterogeneity were:

- EC: ESWT: WBR–Ctr
- HRQoL: CRDQ: WBR–Ctr
- Combined EC + HRQoL: SGRQ:Lb–Ctr and SGRQ: WBR–Ctr
  (Supplementary Material, Table 3)
  Given these results, the selected models were:
- Random-effects models adjusted for age (EC and combined outcomes)
- Random-effects model adjusted for age and BMI (HRQoL)
- Fixed-effects model adjusted for age (EC)

#### Network structure

Nine studies contributed to the EC analysis. Across the three EC outcomes (6MWT, ISWT, and ESWT), 661 outcome-level observations were included in the multivariate model. This figure reflects the cumulative number of participants contributing data across different EC measures and does not represent unique individuals, as some trials reported more than one EC outcome.

Seven studies contributed HRQoL data (CRDQ and/or SGRQ), yielding 278 unique participants in the HRQoL analyses. The combined EC and HRQoL analyses incorporated paired outcome data from these seven studies.

**Fig. 3:**
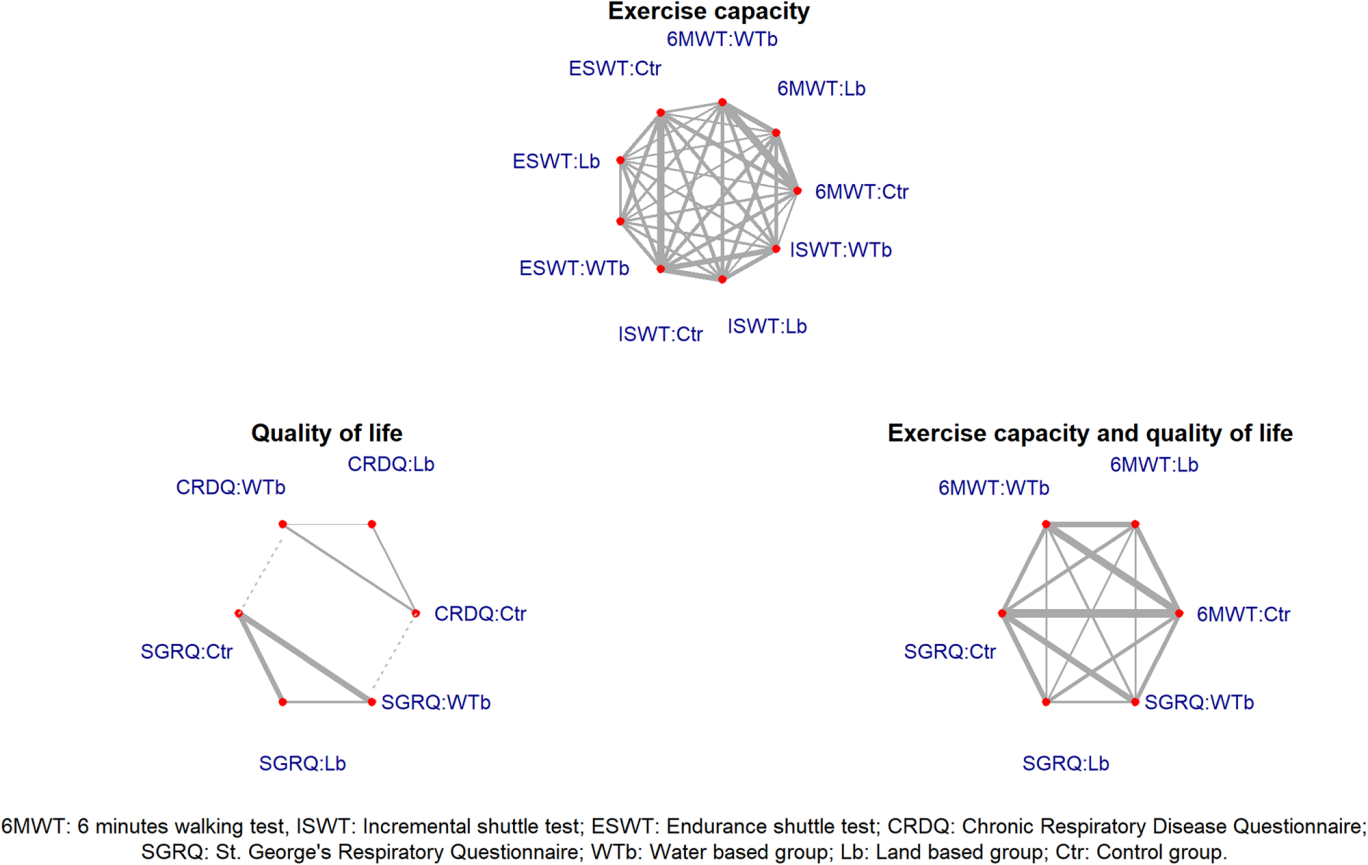
Network constructed for the main outcomes: exercise capacity, HRQoL, and combined exercise capacity and HRQoL. Each node represents an intervention: WBR: Water-based rehabilitation group; Lb: Land-based rehabilitation group; Ctr: Control group. The width of the connecting lines corresponds to the number of trials for each comparison.

The greatest number of comparisons in the EC network involved 6MWT: WBR–Ctr, ESWT: WBR–Ctr, and ESWT:Lb–Ctr. For HRQoL, the most frequent contrasts were SGRQ: WBR–Ctr and CRDQ: WBR–Ctrl. In the combined network, the dominant comparisons were 6MWT: WBR–Ctr and SGRQ: WBR–Ctr (Fig. 3).

#### Ranking of interventions

The ranking analysis indicated that (WBR) achieved the highest P-scores for both EC and combined outcomes, suggesting a superior overall performance compared with land-based and control interventions (Table 2).

**Table 2:** Ranking table of interventions.

Exercise capacity		Quality of life		Exercise capacity and quality of life	
Water based group	>0.999	Land based group	0.763	Water based group	0.955
Land based group	<0.001	Water based group	0.223	Land based group	0.025
Control group	<0.001	Control group	0.014	Control group	0.021

#### Significant interventions

Significant differences were found in EC between the 6MWT:Lb-Ctr, ISWT:Lb-Ctr, 6MWT: WBR-Ctr, ESWT: WBR-Ctr, ISWT: WBR-Ctr, and EC and HRQoL between the SGRQ:Lb-Ctr, 6MWT: WBR-Ctr, and SGRQ: WBR-Ctr with large and significant effect sizes in favor of the WBR or Lb group compared to the control group, without significant effects on HRQoL. Heterogeneity was particularly high in the combined EC and HRQoL model (I² = 98.587%), with EC being the factor that best explained the total variance (R^2^ = 100%) (Table 3).

**Table 3:**
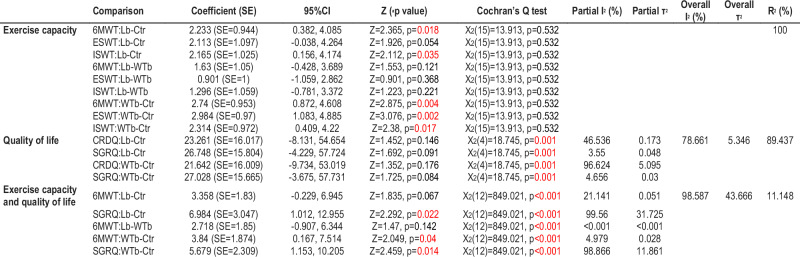
Multivariate pairwise comparison models summary.

*6MWT* 6 min walking test, *ISWT* incremental shuttle test, *ESWT* endurance shuttle test, *CRDQ* chronic respiratory disease questionnaire, *SGRQ* St. George’s respiratory questionnaire, *WTb* water based group, *Lb* land based group, *Ctr* control group.

^a^significant if p < 0.05 (shown in red).

#### Publication bias

The Egger test was not significant for EC (Z = 0.403, p = 0.687) and HRQoL, EC, and HRQoL (Z = 3.567, p < 0.001 and Z = 19.975, p < 0.001, respectively). However, funnel plots demonstrated symmetrical distributions across all comparisons, indicating no publication bias (Supplementary Material, Fig. 1).

#### Summary of certainty of evidence

The GRADE assessment indicated moderate certainty of the effects of both WBR and LBR on EC when compared with control interventions. These effects were consistent across studies and, in most cases, exceeded the established MCID thresholds for the 6MWT, ISWT, and ESWT.

For HRQoL, the certainty of evidence was lower, as the network meta-analysis did not identify significant differences between the intervention and control groups, and moderate inconsistency was present across trials.

For the combined EC and HRQoL outcomes, the certainty of evidence was downgraded to low because of the high heterogeneity and variability in the intervention characteristics among the studies.

No serious concerns regarding publication bias were identified (Table 4).

**Table 4:** Certainty of evidence for the effects of water-based rehabilitation (WBR) and land-based rehabilitation (LBR) compared with control in people with chronic obstructive pulmonary disease (COPD), based on GRADE assessment.

Outcome	No. of studies (participants)	Main comparison	Estimated effect (SMD or MD, 95% CI)	MCID	Direction of effect	Certainty of evidence (GRADE)	Rationale for grading
**Physical performance (6MWT, ISWT, ESWT)**	9 (n = 323)	WBR vs CG	MD 3.58 [1.49, 5.68]	25–33 m (6MWT)	Statistically significant improvement in favour of WBR; clinical interpretation contextualized using established 6MWT MCID thresholds.	Moderate	Downgraded one level for minor inconsistency; no downgrade for imprecision (narrow CI); no evidence of publication bias.
**Physical performance (6MWT, ISWT, ESWT)**	9 (n = 323)	LBR vs CG	MD 3.14 [1.09, 5.19]	25–33 m (6MWT)	Statistically significant improvement in favour of LBR; clinical interpretation contextualized using established 6MWT MCID thresholds.	Moderate	Downgraded one level for inconsistency; robust and homogeneous results (I² = 0%).
**Quality of life (CRDQ, SGRQ)**	7 (n = 278)	WBR vs CG	MD 19.70 [11.71, 27.70] (CRDQ); MD − 24.83 [ − 33.13, −16.53] (SGRQ)	≥0.5 points/item (CRDQ); ≥4 points (SGRQ)	Improvements were observed at study level; however, the network meta-analysis did not demonstrate statistically significant differences versus control.	Moderate	Downgraded one level for moderate inconsistency (I² = 74%); no downgrade for publication bias (symmetric funnel plot).
**Quality of life (CRDQ, SGRQ)**	7 (n = 278)	LBR vs CG	MD 21.24 [13.49, 28.99] (CRDQ); MD − 24.93 [ − 33.23, −16.64] (SGRQ)	≥0.5 points/item (CRDQ); ≥4 points (SGRQ)	Improvements were observed at study level; however, the network meta-analysis did not demonstrate statistically significant differences versus control.	Moderate	Downgraded one level for inconsistency; large and consistent effect sizes.
**Physical performance + Quality of life**	7 (n = 278)	WBR vs CG	MD 3.47 [0.99, 5.94] (6MWT); MD − 4.93 [ − 8.63, −1.23] (SGRQ)	Combined MCID	Significant improvement **in favour of WBR**	Low	Downgraded one level for high inconsistency (I² = 98%); downgraded one level for indirectness (protocol variability).
**Physical performance + Quality of life**	7 (n = 278)	LBR vs CG	MD 3.06 [0.48, 5.64] (6MWT); MD − 5.88 [ − 10.99, −0.78] (SGRQ)	Combined MCID	Significant improvement **in favour of LBR**	Low	Downgraded one level for high inconsistency (I² = 98%); downgraded one level for indirectness.

Certainty of evidence was assessed using the GRADE approach, considering risk of bias, inconsistency, indirectness, imprecision, and publication bias.

*MCID* minimal clinically important difference, *6MWT* 6-Minute Walk Test, *ISWT* incremental shuttle walk test, *ESWT* endurance shuttle walk test, *CRDQ* chronic respiratory disease questionnaire, *SGRQ* St. George’s respiratory questionnaire, *WBR* water-based rehabilitation, *LBR* land-based rehabilitation, *CG* control Group.

**Interpretation of the certainty of evidence**

**• Physical performance: Both WBR and LBR produce clinically meaningful and statistically significant improvements compared with CG, with moderate certainty**.

**• Quality of life: Improvements were observed at study level; however, the network meta-analysis did not demonstrate statistically significant differences compared with CG. Certainty was rated as moderate due to inconsistency**.

**• Combined EC** **+** **QoL outcomes: Significant improvements were observed for both WBR and LBR versus CG, but certainty was downgraded to low due to high heterogeneity and protocol variability**.

**• No relevant publication bias was detected, although the number of available studies for some outcomes remains limited**.

## Discussion

### Interpretation of findings

This systematic review and network meta-analysis examined the comparative effects of WBR, LBR, and control interventions on EC and HRQoL in patients with COPD. However, these findings should be interpreted cautiously. The number of randomized controlled trials including WBR remains limited, which constrains the precision of comparative estimates and precludes definitive conclusions regarding its superiority over LBR. Accordingly, the present results should be understood as indicating the comparative trends and potential clinical advantages of WBR, particularly for EC, rather than as conclusive evidence of greater effectiveness than conventional land-based programs. Rather than interpreting individual trials in isolation, this review integrates evidence across studies using a network meta-analytic framework, allowing for the simultaneous comparison of WBR, LBR, and control interventions across key outcomes. Overall, WBR demonstrated consistent improvements in EC in several trials, particularly those by McNamara et al.^11^, Liu et al.^46^, and de Souto Araujo et al.^42^, which showed changes that exceeded the established MCID thresholds for the 6MWT, ISWT, and ESWT. These findings align with previous evidence from a Cochrane review on aquatic exercise for COPD^55^, which similarly reported meaningful gains in functional performance. The magnitude of improvement was especially pronounced in endurance performance, where hydrostatic pressure and reduced mechanical load likely facilitated greater training intensity and respiratory muscle recruitment.

Regarding HRQoL, improvements have been observed in specific CRDQ and SGRQ domains in several studies. For example, McNamara et al.^11^ reported clinically meaningful improvements in fatigue and dyspnea, whereas Gallo-Silva et al.^45^ and Liu et al.^46^ found significant reductions in SGRQ total scores following WBR. However, these benefits were not consistently observed across all trials, and in the study by de Souto Araujo et al.^42^, HRQoL improvements favored the LBR over the WBR. The variability in HRQoL outcomes likely reflects the heterogeneity of instruments, intervention duration, and sample characteristics, as emphasized in the current PR guidelines^56^.

By applying a network meta-analytic framework, this review integrated both direct and indirect comparisons of the studies. The NMA ranking indicated that WBR achieved the highest P-scores for EC and the combined outcome of EC and HRQoL, whereas LBR ranked highest for overall HRQoL. Age emerged as a significant moderator influencing the treatment effects in several models, suggesting that patient characteristics may play an important role in the response to aquatic or land-based exercise. Despite some domains favoring LBR, WBR frequently demonstrated clinically meaningful improvements compared with control interventions, particularly for endurance-related outcomes.

Taken together, these findings support the consideration of WBR as a complementary component of PR, particularly in individuals with functional limitations or musculoskeletal comorbidities that may hinder land-based training.

### Physiological rationale

WBR offers physiological advantages derived from the unique properties of water, such as buoyancy, viscosity, and hydrostatic pressure, which together support exercise performance in individuals with COPD. Buoyancy reduces the gravitational load and joint stress, enabling individuals with physical comorbidities or deconditioning to exercise with lower perceived effort while achieving greater movement amplitude^16^. Water viscosity provides constant multidirectional resistance, which increases muscle activation and promotes endurance adaptation during repetitive movements.

Hydrostatic pressure exerts a uniform compressive force on the thorax and abdomen, increasing the work of breathing and potentially enhancing the recruitment of respiratory muscles. This mechanism is consistent with the superior gains observed in endurance-based outcomes, particularly in the ESWT, across the trials included in this review. The improved venous return associated with immersion may also contribute to a more efficient cardiac output during moderate-intensity exercise.

Additionally, warm water environments ( ≥ 32 °C) can improve peripheral circulation, reduce muscle stiffness, and attenuate postexercise discomfort, thereby supporting training tolerance and adherence^17^. These combined effects provide a physiological basis for clinically meaningful improvements in EC, as observed in several WBR trials.

### Comparison with prior literature

Previous reviews have reported the beneficial effects of water-based exercise in individuals with COPD; however, the conclusions regarding its superiority over LBR have been inconsistent. A Cochrane review by McNamara et al.^55^ highlighted improvements in exercise tolerance in aquatic programs but noted insufficient evidence to determine whether WBR is more effective than conventional PR. Similarly, a review by Chen et al.^21^ reported improvements in dyspnea, lung function, and EC but did not examine QoL outcomes.

Our findings build upon this earlier evidence by incorporating recent randomized trials, such as those by Liu et al.^46^ and Charususin et al.^12^, and by applying a network meta-analysis framework capable of integrating both direct and indirect comparisons across interventions. This approach allowed us to determine the absolute effects and relative ranking of the WBR, LBR, and control conditions.

Consistent with the previous literature, WBR produced clinically meaningful improvements in EC, particularly in endurance performance, whereas LBR showed benefits predominantly in certain HRQoL domains. The lack of consistent HRQoL improvements across trials contrasts with earlier conclusions, likely reflecting the heterogeneity of HRQoL instruments, intervention duration, and study populations in recent studies. Nevertheless, our results reinforce the current PR guidelines, such as those from the British Thoracic Society^56^, which emphasize structured exercise, whether aquatic or land-based, as a core therapeutic component.

Overall, this review extends prior evidence by demonstrating that WBR is especially effective in improving EC, whereas both WBR and LBR remain superior to usual care in most clinically relevant outcomes.

### Clinical implications

These findings underscore the utility of WBR as a valuable complement or alternative to conventional LBR, particularly for individuals with COPD who experience difficulty performing weight-bearing exercises due to obesity, musculoskeletal comorbidities, or balance impairments. Consistent with our results and prior evidence^56^, clinicians should consider integrating aquatic rehabilitation into individualized PR programs for individuals who may benefit from reduced joint loading and improved-movement tolerance. These findings also support the incorporation of WBR as a complementary modality in established PR programs, particularly for patients with specific functional or musculoskeletal limitations.

WBR demonstrated clinically meaningful improvements in EC, especially in endurance performance, suggesting that aquatic environments may facilitate higher training intensity or longer exercise durations with lower perceived exertion. Although HRQoL improvements were heterogeneous across studies, targeted benefits were observed in fatigue-, dyspnea-, and activity-related domains.

An additional practical advantage is related to the patient experience and adherence. Aquatic settings are often perceived as safer, more comfortable, and less physically demanding, particularly among older adults, which is supported by evidence showing that aquatic environments enhance perceived safety, comfort, and adherence to exercise programs^57^. Prior rehabilitation literature supports this phenomenon, reporting enhanced psychological comfort and higher adherence rates in water-based exercise programs^57^.

Both WBR and LBR are appropriate rehabilitation strategies, and the choice of modality should ultimately depend on patient preference, accessibility to aquatic facilities, comorbidity profiles, and specific functional goals of the patient. Given the substantial heterogeneity observed in the combined outcomes, EC and HRQoL should be monitored as distinct therapeutic targets rather than assuming concurrent improvement.

### Limitations of the review

A major strength of this review was the integration of direct and indirect comparisons through a network meta-analysis, which allowed a comprehensive comparison between WBR, LBR, and control interventions. The evaluation of transitivity, heterogeneity, and consistency strengthened the robustness of the findings, and the use of the MCID thresholds and GRADE provided a clinically meaningful interpretation of the results. Additionally, effect estimates derived from multivariate network models are expressed as model-based coefficients; therefore, clinical interpretation was contextualized using established MCID values from primary studies rather than direct comparison with absolute pooled differences.

However, this study has several limitations that must be acknowledged. First, substantial heterogeneity was observed across studies, particularly in intervention characteristics (e.g., duration, training intensity, water temperature, and supervision) and outcome measures. Quality-of-life outcomes were especially inconsistent because of the use of different instruments (SGRQ vs. CRDQ), incomplete domain reporting, and short intervention durations. Second, many trials included relatively small samples, which increases imprecision and may reduce the ability to detect meaningful between-group differences, a limitation that is well recognized in methodological research^58^.

In addition, inconsistent reporting of key clinical characteristics (e.g., baseline COPD severity, functional status, adherence, and comorbidities) restricted the ability to explore subgroup effects or dose–response relationships of the interventions. Some physiologically relevant outcomes (e.g., respiratory muscle strength, dyspnea scores, and oxygen saturation) have been reported to be inconsistent and could not be included in the quantitative synthesis. Importantly, training intensity, a core variable in exercise prescription and a strong determinant of improvements in EC, has not been systematically reported, preventing its integration as a moderator in the NMA and limiting the interpretation of whether differences in outcomes are attributable to the modality or intensity.

Another methodological constraint was the inconsistent reporting of post-intervention standard deviations and changes in scores across studies. Several trials presented only post-treatment means, percentage changes, or non-parametric summaries, which prevented their immediate integration into the NMA. In some cases, reconstruction of effect sizes was required, and in others, studies could not be included in the specific models. These limitations may have contributed to residual heterogeneity and reduced the precision of pooled estimates.

Therefore, the generalizability of these findings should be interpreted cautiously. The participants in the included trials were willing to engage in aquatic rehabilitation, which may not reflect the broader COPD population. Many individuals with COPD may be reluctant to participate in water-based programs due to concerns about inhaling chlorine fumes, environmental humidity, or perceived risks associated with aquatic exercise. Additionally, access to appropriate aquatic facilities is often limited to urban or hospital-based settings, potentially restricting their feasibility and limiting the routine implementation of WBR in real-world clinical practice.

Finally, several additional limitations should be considered. First, although the literature search covered major biomedical and rehabilitation-focused databases (PubMed, Cochrane Library, PEDro, and a national bibliographic repository), Scopus and the Web of Science were not included. While these databases primarily index overlapping randomized controlled trials already captured by PubMed and Cochrane, their exclusion may have limited the identification of additional eligible studies in our review. However, manual screening of reference lists from the included trials and relevant reviews did not reveal any further randomized controlled trials evaluating WBR in COPD. Therefore, although the risk of missing key evidence is considered low, the findings should be interpreted within the context of the selected databases and the targeted scope of this review.

In addition, the HRQoL analysis was limited to trials that used the CRDQ or SGRQ. Although other instruments, such as the CCQ, CAT, EQ-5D, and SF-36, are widely used, their exclusion was intentional to preserve methodological homogeneity and ensure sensitivity to rehabilitation-related changes. Consequently, the present findings primarily reflect disease-specific QoL domains and may not fully capture broader perceptions of health status.

Finally, although the NMA framework strengthened the comparative evaluation of interventions, the limited number of available trials and variability in methodological quality may have influenced the magnitude and certainty of the pooled estimates. Future high-quality randomized controlled trials with standardized reporting, adequately powered samples, and harmonized intervention protocols are required to confirm these findings.

### Recommendations for future research

Future research should focus on addressing the current methodological limitations and expanding the evidence base for aquatic rehabilitation in patients with COPD. Large-scale multicenter RCTs with standardized and clearly reported intervention protocols are needed to clarify the comparative effectiveness of WBR and LBR across different COPD phenotypes (such as emphysema-predominant, chronic bronchitis-predominant, frequent exacerbators, or phenotypes with prominent systemic or musculoskeletal involvement) and disease severity levels. The consistent use and transparent reporting of validated HRQoL instruments (e.g., CRDQ and SGRQ) and EC tests, including domain-level scores and MCID-based interpretation, will substantially improve the comparability across studies.

Future trials should explore the mechanistic pathways, including respiratory muscle function, dynamic hyperinflation, cardiovascular responses, and the potential mediating role of hydrostatic pressure. Direct comparisons of endurance-focused aquatic protocols with combined or resistance-oriented programs may help identify the most effective training modalities.

Long-term follow-up, real-world adherence assessments, and the inclusion of patient-centered outcomes, such as dyspnea perception, fatigue, functional independence, and daily activity monitoring, are essential for understanding the sustainability and clinical relevance of treatment effects. Although some of the included trials reported respiratory parameters (e.g., dyspnea scores, respiratory muscle strength, SpO₂), and adherence, the reporting was heterogeneous and often incomplete, preventing their inclusion in the quantitative synthesis. Incorporating cost-effectiveness analyses and implementation-focused designs would further support the translation of WBR into routine PR practices.

## Conclusion

WBR was associated with clinically meaningful improvements in EC in individuals with COPD and may represent an effective alternative or complement to land-based exercise programs. Benefits were most evident in endurance performance, whereas the effects on HRQoL were more variable. Both active modalities outperformed usual care, although heterogeneity and limited sample sizes warrant cautious interpretation of the results.

## Supplementary information

Supplementary material

Supplementary information

## Data Availability

Data are provided within the manuscript or supplementary information files.
