# Supplementary material for: Comparative effectiveness of water-based versus land-based rehabilitation in COPD: a systematic review and network meta-analysis of randomized controlled trials
Source: NPJ Prim Care Respir Med. 2026 Apr 11;36:52. doi: 10.1038/s41533-026-00503-8 (PMC13427819; doi:10.1038/s41533-026-00503-8)

**Supplementary material. Table 1**: Confounding variables effect.

|  |  | **Coefficient (SE)** | **95%CI** | **Z (^a^p value)** |
| --- | --- | --- | --- | --- |
| **Exercise capacity** | Age | -0.039 (SE=0.02) | -0.078, 0 | Z=-1.971, p=0.049 |
|  | Body Mass Index | -0.02 (SE=0.043) | -0.104, 0.064 | Z=-0.458, p=0.647 |
|  | Female/male ratio | 0.064 (SE=0.278) | -0.481, 0.608 | Z=0.229, p=0.819 |
| **Quality of life** | Age | -0.619 (SE=0.242) | -1.093, -0.144 | Z=-2.557, p=0.011 |
|  | Body Mass Index | 1.109 (SE=0.459) | 0.209, 2.009 | Z=2.414, p=0.016 |
|  | Female/male ratio | -1.01 (SE=1.635) | -4.215, 2.195 | Z=-0.618, p=0.537 |
| **Exercise capacity and quality of life** | Age | -0.058 (SE=0.042) | -0.14, 0.023 | Z=-1.396, p=0.163 |
|  | Body Mass Index | -0.006 (SE=0.08) | -0.162, 0.15 | Z=-0.075, p=0.94 |
|  | Female/male ratio | 0.109 (SE=0.515) | -0.901, 1.118 | Z=0.211, p=0.833 |

SE: Standard error; 95%CI: 95% confidence interval. ^a^significant if p<0.05 (shown in red).

**Supplementary material. Table 2:** Assessment of heterogeneity and consistency.

|  | **Model** | **AIC** | **^a^p value** |
| --- | --- | --- | --- |
| **Hetergeneity test** | | | |
| **Physical performance** | Full | 85 |  |
|  | Reduced | 33 | X^2^(9)=7, p>0.999 |
| **Quality of life** | Full | 41 |  |
|  | Reduced | 85 | X^2^(4)=56, p<0.001 |
| **Physical performance and quality of life** | Full | 59 |  |
|  | Reduced | 1147 | X^2^(5)=1110, p<0.001 |
| **Consistency test** | | | |
| **Physical performance** | Full | 139 |  |
|  | Reduced | 85 | X^2^(38)<0.001, p>0.999 |
| **Quality of life** | Full | 61 |  |
|  | Reduced | 41 | X^2^(10)<0.001, p>0.999 |
| **Physical performance and quality of life** | Full | 81 |  |
|  | Reduced | 59 | X^2^(16)<0.001, p>0.999 |

AIC: Akaike information criterium. ^a^significant if p<0.05 (shown in red).

**Supplementary material. Table 3:** Pairwise direct contribution table.

| **Physical performance** | | **Quality of life** | | **Physical performance and quality of life** | |
| --- | --- | --- | --- | --- | --- |
| **Comparison** | **Contribution (%)** | **Comparison** | **Contribution (%)** | **Comparison** | **Contribution (%)** |
| 6MWT:Lb-WTb | 3.23 | CRDQ:Lb-Ctr | 0 | 6MWT:Lb-WTb | 0.01 |
| 6MWT:Lb-Ctr | 1.53 | CRDQ:WTb-Ctr | 42.09 | 6MWT:Lb-Ctr | 0.06 |
| 6MWT:WTb-Ctr | 3.08 | CRDQ:WTb-Ctr | 51.71 | 6MWT:WTb-Ctr | 0.04 |
| 6MWT:Lb-WTb | 2.75 | SGRQ:Lb-Ctr | 0.04 | 6MWT:Lb-WTb | 0 |
| 6MWT:WTb-Ctr | 3.33 | SGRQ:WTb-Ctr | 0.53 | 6MWT:WTb-Ctr | 0.01 |
| 6MWT:Lb-Ctr | 0.93 | SGRQ:WTb-Ctr | 3.25 | 6MWT:Lb-Ctr | 0.02 |
| 6MWT:WTb-Ctr | 0.15 | SGRQ:Lb-Ctr | 0.68 | 6MWT:WTb-Ctr | 0.01 |
| 6MWT:Lb-Ctr | 3.03 | SGRQ:WTb-Ctr | 0.99 | 6MWT:Lb-Ctr | 0 |
| 6MWT:WTb-Ctr | 0.74 | SGRQ:Lb-Ctr | 0.15 | 6MWT:WTb-Ctr | 0 |
| 6MWT:WTb-Ctr | 0.47 | SGRQ:WTb-Ctr | 0.56 | 6MWT:WTb-Ctr | 0 |
| 6MWT:Lb-WTb | 0.01 |  | NA | 6MWT:Lb-WTb | 0 |
| ESWT:Lb-WTb | 1.8 |  | NA | SGRQ:Lb-Ctr | 55.94 |
| ESWT:Lb-Ctr | 0.66 |  | NA | SGRQ:WTb-Ctr | 21.19 |
| ESWT:WTb-Ctr | 3.56 |  | NA | SGRQ:WTb-Ctr | 2.23 |
| ESWT:WTb-Ctr | 18.45 |  | NA | SGRQ:Lb-Ctr | 5.26 |
| ESWT:Lb-Ctr | 0.01 |  | NA | SGRQ:WTb-Ctr | 2.39 |
| ESWT:WTb-Ctr | 33.9 |  | NA | SGRQ:Lb-Ctr | 9.5 |
| ISWT:Lb-WTb | 0.74 |  | NA | SGRQ:WTb-Ctr | 3.33 |
| ISWT:Lb-Ctr | 0.42 |  | NA |  | NA |
| ISWT:WTb-Ctr | 5.99 |  | NA |  | NA |
| ISWT:Lb-Ctr | 6.03 |  | NA |  | NA |
| ISWT:WTb-Ctr | 0.46 |  | NA |  | NA |
| ISWT:Lb-Ctr | 3.19 |  | NA |  | NA |
| ISWT:WTb-Ctr | 2.22 |  | NA |  | NA |
| ISWT:Lb-WTb | 3.31 |  | NA |  | NA |
| 6MWT:Lb-WTb | 3.23 |  |  |  |  |
| 6MWT:Lb-Ctr | 1.53 |  |  |  |  |
| 6MWT:WTb-Ctr | 3.08 |  |  |  |  |

6MWT: 6 minutes walking test, ISWT: Incremental shuttle test; ESWT: Endurance shuttle test; CRDQ: Chronic Respiratory Disease Questionnaire; SGRQ: St. George’s Respiratory Questionnaire; WTb: Water based group; Lb: Land based group; Ctr: Control group. Values higher than 10% are shown in red.

**Supplementary material. Figure 1:** Publication bias pairwise-comparisons funnel plots.


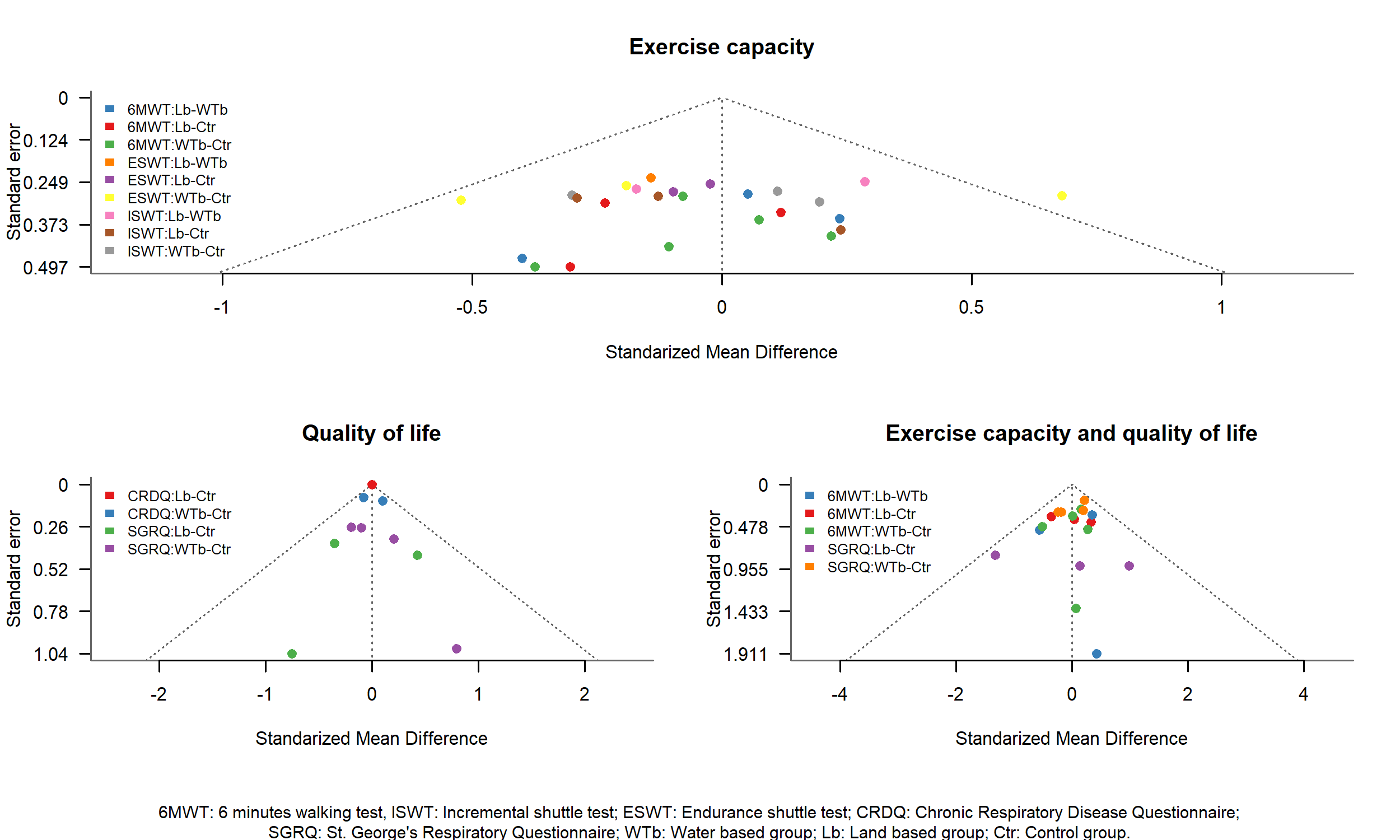

Supplement: Supplementary file 1 — Supplementary material [file 41533_2026_503_MOESM1_ESM.docx]
