# Supplementary material for: Comparative effectiveness of water-based versus land-based rehabilitation in COPD: a systematic review and network meta-analysis of randomized controlled trials
Source: NPJ Prim Care Respir Med. 2026 Apr 11;36:52. doi: 10.1038/s41533-026-00503-8 (PMC13427819; doi:10.1038/s41533-026-00503-8)
Supplement: Supplementary file 2 — Supplementary information [file 41533_2026_503_MOESM2_ESM.docx]

**Supplementary Material. Search Strategy Details by Database**

| **Database** | **Search Syntax (Boolean + keywords)** | **Filters Applied** | **Records Retrieved** |
| --- | --- | --- | --- |
| **PubMed** | ("COPD"[MeSH] OR "Chronic Obstructive Pulmonary Disease") AND (water OR aqua* OR hydro* OR "aquatic therapy" OR "hydrotherapy") AND ("pulmonary rehabilitation" OR exercise* OR physiotherapy) AND (land OR "conventional therapy" OR "usual care") | Humans, Adults, RCTs, English/French/Spanish/Portuguese | 124 |
| **Cochrane** | COPD AND (aquatic OR water OR hydrotherapy) AND rehabilitation | Trials only; language filters applied according to eligibility criteria | 45 |
| **BnL** | ("Chronic Obstructive Pulmonary Disease") AND (aqua* OR hydrotherapy) AND (exercise OR rehabilitation OR treatment) | Peer-reviewed articles, Adults only | 46 |
| **PEDro** | Title/Abstract: COPD AND (water OR hydrotherapy OR aquatic) | Clinical trials only | 17 |

This supplementary material provides a complete overview of the search strategies used for each database included in this systematic review. All databases were searched from their inception to the final search date (July 13, 2025). No earlier date limits were applied and structured using Boolean operators and Medical Subject Headings terms when applicable. No automated database filters were applied (e.g., study design, population, language, or age). Instead, all retrieved records were manually screened to identify randomized controlled trials (RCTs) involving adult humans. Articles published in English, French, Spanish, or Portuguese were considered to be eligible.
